# Acetaminophen cytotoxicity is ameliorated in a human liver organotypic co-culture model

**DOI:** 10.1038/srep17455

**Published:** 2015-12-03

**Authors:** Leonard J. Nelson, Maria Navarro, Philipp Treskes, Kay Samuel, Olga Tura-Ceide, Steven D. Morley, Peter C. Hayes, John N. Plevris

**Affiliations:** 1 Department of Hepatology, Hepatology Laboratory, University of Edinburgh, Edinburgh, UK; 2 Scottish National Blood Transfusion Service (SNBTS); Cell Therapy Research Group, Scottish Centre for Regenerative Medicine, University of Edinburgh, UK; 3 Department of Pulmonary Medicine, Hospital Clínic-Institut d’Investigacions Biomèdiques August Pi i Sunyer (IDIBAPS); University of Barcelona. Centro de Investigación Biomédica en Red de Enfermedades Respiratorias, Spain

## Abstract

Organotypic liver culture models for hepatotoxicity studies that mimic *in vivo* hepatic functionality could help facilitate improved strategies for early safety risk assessment during drug development. Interspecies differences in drug sensitivity and mechanistic profiles, low predictive capacity, and limitations of conventional monocultures of human hepatocytes, with high attrition rates remain major challenges. Herein, we show stable, cell-type specific phenotype/cellular polarity with differentiated functionality in human hepatocyte-like C3A cells (enhanced CYP3A4 activity/albumin synthesis) when in co-culture with human vascular endothelial cells (HUVECs), thus demonstrating biocompatibility and relevance for evaluating drug metabolism and toxicity. In agreement with *in vivo* studies, acetaminophen (APAP) toxicity was most profound in HUVEC mono-cultures; whilst in C3A:HUVEC co-culture, cells were less susceptible to the toxic effects of APAP, including parameters of oxidative stress and ATP depletion, altered redox homeostasis, and impaired respiration. This *resistance* to APAP is also observed in a primary human hepatocyte (PHH) based co-culture model, suggesting bidirectional communication/stabilization between different cell types. This simple and easy-to-implement human co-culture model may represent a sustainable and physiologically-relevant alternative cell system to PHHs, complementary to animal testing, for initial hepatotoxicity screening or mechanistic studies of candidate compounds differentially targeting hepatocytes and endothelial cells.

Development of *organotypic* human hepatic models that more closely resemble liver function is highly desirable for pre-clinical assessment of new candidate compounds in drug development. The need for improved models for hepatotoxicity screening or mechanistic studies is underscored by the high attrition rate of drugs due to liver toxicity. This is partly due to the fact that drug metabolism in animals often does not reflect that seen in humans^1^; whilst current *in vitro* models including monocultures of human hepatic cell lines, are limited in their ability to accurately and consistently represent *in vivo* drug metabolism pathways^2^.

Hepatic models using primary human hepatocytes (PHHs) are currently preferred for *in vitro* drug testing; however, they have major limitations for drug safety studies. PHHs are scarce and expensive, with considerable batch variation in hepatic functionality^2^. *In vitro* cultures of PHHs have restricted growth activity and lifespan, and undergo early phenotypic alterations. Wide variations in functional activities, especially CYP450 levels/magnitude of CYP450 induction have been reported between human hepatocyte populations. Crucially, all CYP450s are not similarly maintained, dedifferentiating with time in culture^3^. This implies that such differences in stability of individual CYP450s in culture could result in an artificial culture phenotype that does not reflect the donor phenotype^4^. Moreover, upon isolation, PHHs are in a state of ‘pre-apoptotic cell stress’ signifying that they are already committed to death after the isolation process^5^. This raises issues on the integrity of PHHs used for modeling metabolic processes, including hepatoxicity studies. Therefore development of more practical, sustainable, and stable organotypic alternatives would seem appropriate for drug toxicity testing. Indeed, co-cultures of hepatocytes with other cell types are now considered highly promising alternatives for toxicological studies^2^. Co-culture of hepatic- with non-parenchymal or non-hepatic-derived stromal cells can improve liver-specific functions, cell survival and stabilize hepatic phenotype *in vitro*^6^,^7^. Given the inherent phenotypic instability of PHHs, alternative co-culture-based systems utilizing hepatocyte-like cells with stromal cell support, may provide more *in vivo*-like cues providing functional and metabolic stability, recognized as key for correct interpretation of *in vitro* toxicity data^2^. In principle, such systems may be used as a bridge between animal models and humans as the first step in risk assessment^8^.

Practical alternatives for PHHs currently used in drug testing include human hepatic cell lines such as HepaRG, Huh7 and HepG2 cell lines, although with incomplete metabolic profiles compared with primary hepatocytes. Indeed, CYP450 enzymes responsible for catalyzing acetaminophen oxidation to NAPQI in human liver are either absent (CYP2E1), or at low (CYP3A4) levels in both HepG2 and C3A cells (unpublished observations). However, recent studies suggest that CYP3A4 is in fact the major enzyme form catalysing APAP in humans^9^,^10^,^11^. The C3A cell line is a clonal derivative of the widely used hepatoblastoma-based HepG2 cell line, selected for its more differentiated hepatic phenotype^12^. The utility of C3A hepatocyte-like cells is shown by their implementation in a commercial bioartificial liver system (vitaltherapies.com/elad/technology/)^12^. Previously, we demonstrated that preconditioning of C3A cells with appropriate trophic support significantly increases their metabolic capacity and efficacy for bioartificial therapy; and have used C3As to accurately model non-alcoholic fatty liver disease *in vitro*^13^. Recently, co-culture of iPSC-derived hepatocytes with endothelial cells has demonstrated the ability of the latter to prolong the viability of hepatocyte-like cells and enhance their functional capabilities^14^.

To more accurately reproduce *in vitro* the mechanisms involved in drug metabolism *in vivo*, hepatocytes require interaction with non-parenchymal cells, including endothelial cells. It has been shown that hepatocyte/endothelial co-culture improved hepatic protein synthesis and detoxification activity *in vitro*^15^,^16^,^17^,^18^; whilst co-culture with human liver sinusoidal endothelial cells (LSECs) highlight the importance of heterotypic cell interactions for bidirectional stabilization^19^. LSECs are a unique endothelial cell type due to open fenestrations and strong capacity for endocytosis, characteristics not observed in other ECs. However, although LSECs represent the ideal stromal support cell for *in vitro* modeling, these cells are not readily available, difficult to isolate and present an unstable phenotype *in vitro*^20^,^21^,^22^,^23^.

As alternatives to LSECs, several studies have used diverse co-culture systems (e.g. rat hepatocytes/human aortic endothelial cells; rat hepatoma cell line (Fao)/HUVECs; HepG2/HUVECs) which also demonstrate enhanced differentiated functionality, through direct physical cell-cell interactions/trophic support and bidirectional cross-talk between cell types - in drug studies^16^,^21^ and liver organoid formation *in vitro*^17^,^24^,^25^,^26^. Moreover, emerging evidence suggests liver endothelial cells as well as extra-hepatic tissues might represent a direct target of the cytotoxic effect of acetaminophen (APAP)^27^,^28^.

To date, the vast majority of *in vitro* models studying APAP (or drug) toxicity use monocultures of hepatocytes, which ignore the potentially crucial contributions of endothelial cells in maintaining hepatocyte differentiation and modulating drug metabolism. APAP is considered a model (intrinsic) hepatotoxin, and is a major cause of acute liver failure in USA and Europe; making the study of APAP toxicity both clinically relevant and experimentally convenient. Indeed, studies with model toxicants such as APAP have helped to define the roles that chemical stress and drug bioactivation have in the various biological outcomes that may be triggered by chemically reactive metabolites^8^,^29^.

We hypothesized that a heterotypic co-culture system may represent an improved, physiologically-relevant *in vitro* model for assessing aspects of drug-induced liver injury (DILI); using APAP as a model toxicant, which has well-studied mechanisms of hepatotoxicity and is used for establishing the usefulness of *in vitro* models^30^.

Hence, the aims of this study were to: (i) Develop a phenotypically-stable human co-culture system using C3A hepatocyte-like cells and endothelial cells (HUVECs) as sustainable surrogates for PHH and LSECs; (ii) Demonstrate utility through assessment of form, phenotypic and functional stability of co-cultures, compared with mono-cultures; and (iii) To evaluate susceptibility to a prototypical hepatotoxin (APAP) on cytotoxicity parameters in mono- and co-culture systems.

## Materials and Methods

### C3A and HUVEC mono- and co-cultures

Human hepatoblastoma C3A cells (C3As; American Type Culture Collection CRL-10741) were maintained on polystyrene with Minimum Essential Medium Eagle +10% FBS (MEME+; Sigma-Aldrich). Human Umbilical Vein Endothelial Cells (HUVECs; Lonza) were maintained in EGM-2 medium (EGM-2; Lonza). To establish conditions for phenotypically stable co-cultures, C3As and HUVECs were first cultured separately in both MEME+ and EGM-2 for 3 days before assessment of various phenotypic and morphological criteria. Subsequently, C3As and HUVECs were seeded in EGM-2 in 6-well, 12-well, 96-well plates, at an initial density of 21,000 cells/cm^2^ in mono-cultures and 21,000 cells/cm^2^ at a ratio of 1:1 in C3A:HUVEC co-cultures, unless otherwise indicated. For co-cultures, HUVECs were seeded 4 h before the addition of C3As to allow them to adhere to tissue culture plates. Cells were then cultured for 3 days before the assessment of biocompatibility or the application of treatments. For APAP treatment, mono- and co-cultures were incubated for 24 hr in culture medium containing 0, 5, 10 or 20 mM APAP for 24 hr (0 mM APAP represents untreated control). In some experiments, following treatment with APAP, cells were washed twice in PBS and then cultured in EGM-2 containing 5 mM N-acetyl cysteine (NAC), or EGM-2 alone, for a further 24 hr to assess recovery from APAP treatment.

### Immunocytochemistry

Cells were fixed in 4% paraformaldehyde (Sigma-Aldrich) for 30 min at RT and further treated for 1 h at RT with 0.1% Triton X-100 in PBS before blocking with PBS containing 5% BSA for 30 min. For detection of endothelial CD31 and hepatic EpCAM specific cell markers, cells were incubated with rabbit-anti-human CD31 (eBioScience, Hatfield, UK) (1:100) and/or directly conjugated mouse anti-human APC-EpCAM (Biolegend, London, UK) (1:100) at 4 °C overnight. For CYP3A4 detection, cells were incubated with primary rabbit-anti-human Cytochrome P450 Enzyme CYP3A4 Antibody (Chemicon, Millipore) (1:800) and Rhodamine phalloidin (Life Technologies) (1:100) for 90 min. In both cases, cells were then treated with appropriate secondary anti rabbit- or anti-mouse-Alexa Fluor 488 (Life Technologies) for 45 min at RT before being stained with DAPI (Sigma Aldrich) at 1/5000 for 5 min. Cell morphology was assessed using Zeiss Axio-Observer A1 microscope, and separate fluorescent images were captured with a Zeiss AxioCam MRm camera (Carl Zeiss, Germany) and merged using ImageJ (1.46r) software.

### Assessment of hepatic functionality

Albumin synthesis was measured in cell culture supernatants on day 3 after 24 h in serum- free EGM-2 medium using an Albumin Blue 580 Fluorescence Assay as previously described^31^. Data was normalized to the number of viable C3As seeded (day 0). CYP3A4 enzyme activity was assessed using a non-lytic luminescence assay (P450-Glo, Promega) following the manufacturer’s instructions.

### Mitochondrial function and cell viability following acetaminophen exposure

In two sets of parallel experiments following APAP exposure cell viability was determined with the CellTiter-Glo Luminescent Cell Viability Assay (cellular ATP content; Promega, TB288) or with the Mitochondrial ToxGlo™ Assay (Promega, TM357) according to the manufacturer, to differentially evaluate both cell membrane integrity and mitochondrial function in mono- or co-cultures, without (control), or with 5 10, and 20 mM APAP for 24 h.

### Live/Dead fluorescent staining

Viability was determined using a LIVE/DEAD® Viability/Cytotoxicity Kit (Life technologies).

### Parameters of cellular redox potential (pyruvate/lactate) and nitric oxide (NO) levels

Total Nitric Oxide (NO) concentration was measured in cell culture supernatants by ELISA using a Total Nitric oxide and Nitrate/Nitrite Parameter assay kit (R&D systems). Extracellular levels of lactate and pyruvate were measured as previously described^12^. Where indicated, data was normalized to total cellular protein as measured using a bicinchoninic acid assay (BCA assay, Pierce) according to the manufacturer’s instructions.

### Statistical Analysis

All cell culture data were obtained from independent mono- or co-culture experiments (triplicate biological replicates; n = 3), using triplicate wells (technical replicates) within a single experiment. GraphPad Prism 5 software (GraphPad Software Inc, USA) was used for statistical analysis. Results are presented as mean ± standard error of the mean (SEM). A two-tailed unpaired student’s t test was used to test significance between individual data sets as indicated. A p-value < 0.05 was considered statistically significant.

## Results

### Biocompatibility of C3A: HUVEC co-cultures

**Figure 1:**
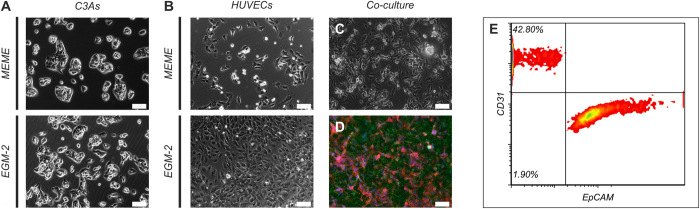
Morphology of C3As and HUVECs in mono- and co-culture. Phase contrast images of (**A**) C3As cultured in MEME+ and EGM-2; (**B**), HUVECs cultured in MEME+ and EGM-2; or (**C**) C3A:HUVEC co-cultures in EGM-2 at an initial plating ratio of 1:1. (**D**) Fluorescence image of C3A:HUVEC co-cultures in EGM-2 stained with CD31 endothelial cell marker (green; identifies HUVECs), EpCAM epithelial cell marker (red; identifies C3A hepatocyte-like cells) and counterstained with DAPI (blue). Scale bars = 100 μm. (**E**) Flow cytometry analysis of disaggregated C3A:HUVEC co-cultures stained with CD31 endothelial cell marker and EpCAM epithelial cell marker, which showed average co-culture ratios of 37.3 ± 5.4% CD31+ endothelial cells and 56.6 ± 4.5% EpCAM+ hepatocyte-like cells, following three days of culture in EGM-2.

**Figure 2:**
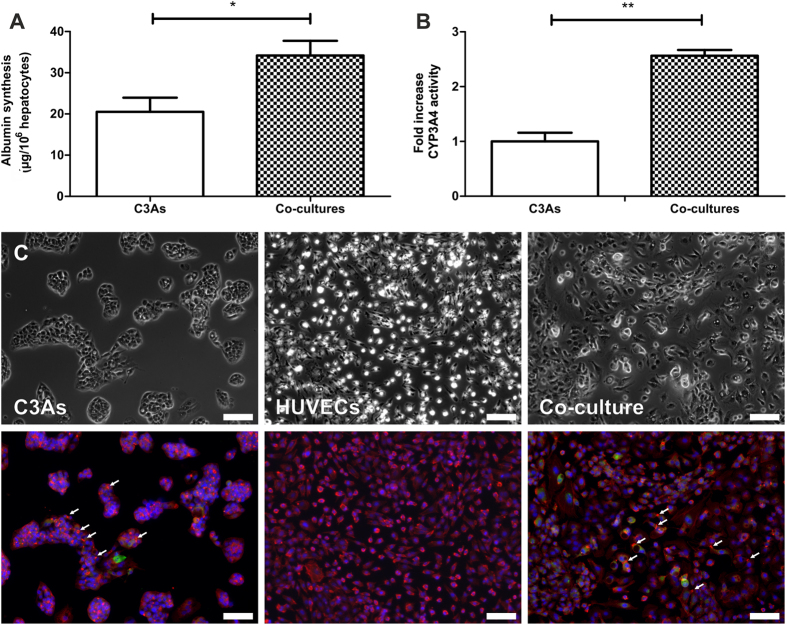
Hepatic functionality of C3As in mono- and co-culture. Determination of (**A**) albumin synthesis and (**B**) CYP3A4 activity in C3A monocultures and C3A:HUVEC co-cultures. Data is expressed as the mean ± SEM (n = 3), *p < 0.05, **p < 0.01. (**C**) Phase contrast and fluorescence images of C3A and HUVEC monocultures or C3A:HUVEC co-cultures stained with antibodies specific for F-actin (red), CYP3A4 (green) and counterstained with DAPI (blue). Arrows indicate examples of F-actin bands surrounding morphological bile canalicular structures confirming the presence of differentiated cellular polarity seen during albumin production. Scale bars = 100 μm.

C3As and HUVECs mono-cultures were evaluated by phase-contrast imaging in mono- and co-culture in either hepatic (MEME+) or endothelial (EGM-2) media. Whilst characteristic epithelial morphology of C3As was retained in both MEME+ and EGM-2 media after 3 days of culture, the characteristic endothelial ‘cobblestone’ morphology of HUVECs was absent in MEME+, but present in EGM-2 medium (Fig. 1A,B). Phase contrast and immunostaining images of co-cultures in EGM-2 medium showed morphological stability of both cell types and self-organizational properties (Fig. 1C,D). Flow cytometry analysis of disaggregated C3A:HUVEC co-cultures stained with CD31 endothelial cell marker and EpCAM epithelial cell marker, which showed average co-culture ratios of 37.3 ± 5.4% CD31+ endothelial cells and 56.6 ± 4.5% EpCAM+ hepatocyte-like cells, following three days of culture in EGM-2 (Fig. 1E). We also show that both C3A mono-cultures and C3A:HUVEC co-cultures in EGM-2 maintain bile-canalicular structures; evidenced as punctate staining of F-actin bands surrounding these structures (Fig. 2C), indicative of bile- canaliculae [phalloidin-staning]. This confirms the presence of specific hepatocyte phenotypic features, namely the differentiated cellular polarity and albumin production. Similar morphological/phenotypic profiles have been reported in a PHHs:HUVEC organotypic co-culture system^18^.

### Hepatic functionality

Hepatic-specific function was compared between C3A mono- and co-cultures, with regard to albumin synthesis and CYP3A4 activity. C3As showed significantly greater albumin synthesis when co-cultured with HUVECs (p = 0.011 vs C3A mono-culture) (Fig. 2A). Additionally, there was a significant, 2.6 fold increase (p = 0.001 vs C3A mono-culture) in CYP3A4 activity in co-cultures (Fig. 2B). Higher CYP3A4 fluorescent intensity was observed in co-cultures compared to C3A mono-cultures (Fig. 2C). Hepatic functionality/metabolic competency suitable for drug metabolism/toxicity studies, in our co-culture system compared well with different *in vitro* culture systems utilizing principally hepatocytes/and or endothelial cells (SI Table S1).

### Multiple metabolic parameters of acetaminophen cytotoxicity

#### Cell viability

**Figure 3:**
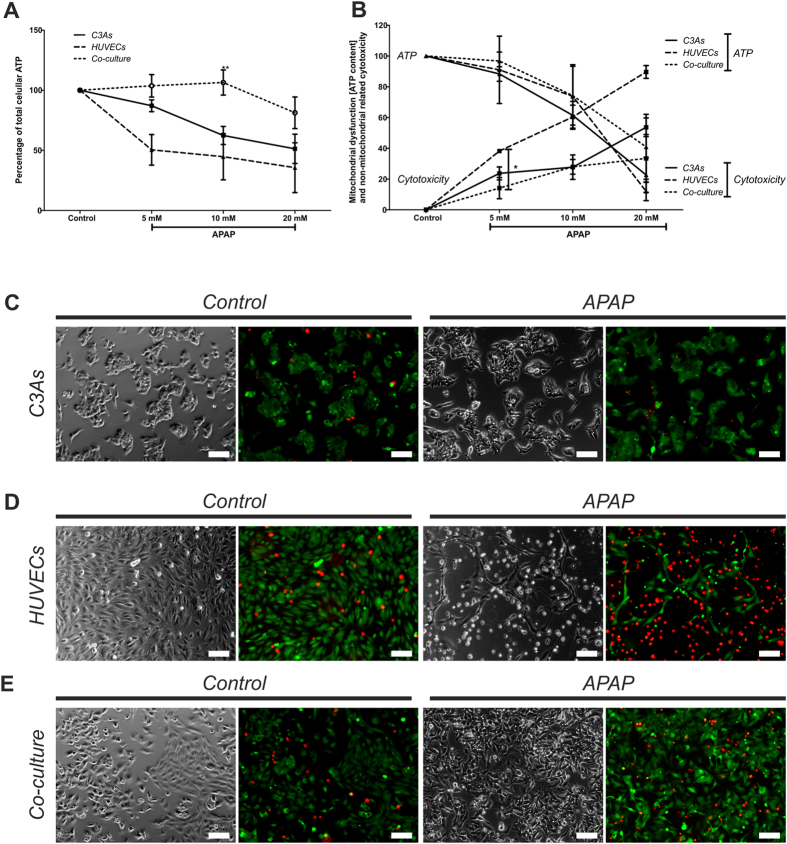
Mitochondrial dysfunction and cell viability in C3A and HUVEC mono- and co-cultures following 24 h APAP exposure. Assessment of (**A**) cell viability by measurement of total intracellular ATP content in C3A and HUVEC monocultures or C3A:HUVEC co-cultures and (**B**) mitochondrial dysfunction by measurement of ATP generated following 90 minutes incubation with D-(+)-galactose, following exposure to 0 mM (untreated control), 5 mM, 10 mM, or 20 mM APAP for 24 hr. Data is expressed as the percentage mean ± SEM (n = 3), *P < 0.05, **p < 0.01. Phase contrast and fluorescence images of (**C**) C3A monocultures; (**D**) HUVEC mono cultures; or (**E**) C3A:HUVEC co-cultures subjected to live (bright green)/dead (bright red) viability staining, either following treatment with 0 mM (untreated control) or 10 mM APAP for 24 hr. Red and green stained images were merged using ImageJ 1.46r. Magnification ×10; Scale bars = 100 μm.

To assess APAP cytotoxicity after 24 h, cell viability was measured using an ATP depletion (endpoint) viability assay in both control (untreated) and treated mono- and co-cultures (Fig. 3A). In mono-cultures, APAP caused a dose-dependent decrease in viability at all doses. In contrast, at low (5 mM) to intermediate (10 mM) APAP, we observed significantly greater ATP levels in co-culture compared with mono-cultures (eg at 10 mM; p = 0.007), indicating a cytoprotective effect in this model system. This phenomenon was evident even at 20 mM APAP, with 80% of co-cultured cells viable.

#### Multiplex assay

The Mitochondrial ToxGlo Assay was also used to provide a differential measurement of biomarkers associated with changes in cell membrane integrity (cytotoxicity) over 24 h, and cellular ATP levels (mitochondrial dysfunction), relative to vehicle-treated control during short exposure periods (Fig. 3B). Data shows significant differences between mono- and co-cultures.

#### Cytotoxicity

Following 5 mM APAP for 24 h, cytotoxicity was significantly greater in C3A monocultures compared with co-cultures (p = 0.026). HUVECs mono-cultures were highly sensitive at all doses tested (p < 0.05). With intermediate dose (10 mM) APAP, C3A mono- and co-cultures exhibited a similar cytotoxicity profile; whilst at 20 mM, co-culture conferred resistance to APAP cytotoxicity compared with both C3A mono-cultures (Fig. 3B).

#### Mitochondrial dysfunction

C3A and HUVECs in mono- and co-cultures were incubated with D-galactose for 90 min. Here, cells must use oxidative phosphorylation to generate ATP and are therefore more responsive to mitochondrial perturbation (‘galactose ATP’). This provides information on the capacity of the cells to produce mitochondrial ATP after APAP exposure. Figure 3B shows a dose-dependent reduction in ATP production for both mono- and co-cultures. Although no significant differences were observed, C3As followed by HUVEC mono-cultures showed a more pronounced drop in mitochondrial ATP than in co-cultures.

#### Cytotoxicity phenotype

Live/Dead staining (Fig. 3C–E) of cultures following 10 mM APAP, revealed an apparently greater cytotoxic effect on HUVEC mono-cultures compared with C3A mono-cultures. In contrast, co-cultures appeared to confer resistance to APAP-induced HUVEC damage (less red staining). Indeed, cell toxicity, measured as ATP depletion (Fig. 3A), correlate with these phenotypic observations.

### Parameters of cellular redox potential

#### Pyruvate/Lactate ratio

**Figure 4:**
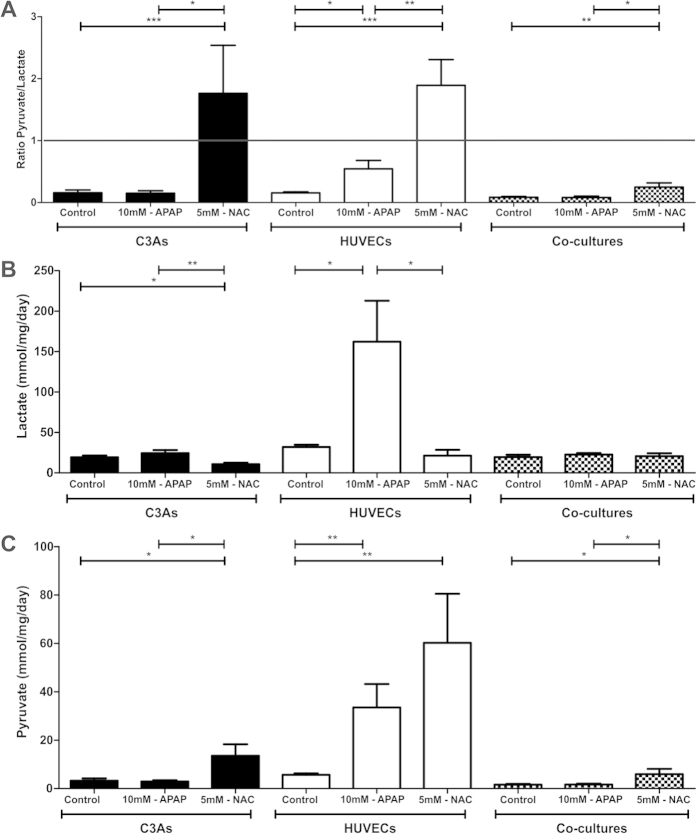
Pyruvate/lactate ratios in C3A and HUVEC mono- and co-cultures treated with APAP followed by NAC. Determination of (**A**) pyruvate/lactate ratios or individual values for (**B**) lactate and (**C**) pyruvate in C3A (black bars) and HUVEC (white bars) mono-cultures or C3A/HUVEC co-cultures (patterned bars) treated with 10 mM-APAP for 24 hr followed by culture in EGM-2 for 24 hr (10 mM-APAP), before cells were treated with 10 mM-APAP for 24 hr followed by 5 mM-NAC for a final 24 hr (5 mM-NAC), or cultured in EGM-2 for both periods (control). Pyruvate/lactate ratios and values for individual metabolites are displayed as the mean ± SEM (n = 3); *p < 0.05, **p < 0.01, ***p < 0.001.

Next, we measured parameters of cellular redox potential (anaerobic and aerobic metabolism) and the effect of the antioxidant N-acetyl cysteine (NAC) following exposure to 10 mM APAP in mono- and co-cultures. Figure 4A shows that the Pyruvate/Lactate ratio in both mono-cultures of C3As and co-cultures was not significantly influenced by exposure to 10 mM APAP; favoring anaerobic metabolism (P/L ratio < 1). Compared with both control and 10 mM APAP-treated cells, NAC administration switched both C3As and HUVECs mono-cultures to aerobic metabolism (P/L ratio > 1), but not in co-cultures (Fig. 4A).

Individual measurements of extracellular lactate (Fig. 4B) and pyruvate (Fig. 4C) concentrations however, showed clear differences between mono- and co-cultures. Lactate production has recently been demonstrated as a global marker of oxidative stress and sub-lethal toxicity in human hepatoma HepaRG cells, renal tubules and fibroblasts, in response to a range of toxicants^32^. Figure 4B shows that APAP treatment led to a slight increase in lactate production by C3As *vs* untreated controls, whereas in APAP-treated HUVECs, lactate production increased significantly (5-fold) compared with HUVEC controls. NAC treatment significantly reduced lactate levels in both C3As (p = 0.008) and HUVECs (p = 0.028). No significant changes were observed in in co-cultures, suggesting reduced oxidative stress in this setting.

Finally, Fig. 4C shows that pyruvate release in C3A mono- or co-cultures, was not significantly changed by APAP exposure. Subsequent addition of NAC however, resulted in a significant increase in treated C3A mono-cultures (p = 0.02) and co-cultures (p = 0.04). In HUVECs, pyruvate release was significantly increased (6-fold) in APAP-treated cells *vs* control (p = 0.009); whilst subsequent treatment with NAC further increased pyruvate levels. This effect is not observed in isolated mouse hepatocytes, where pyruvate-supported respiration is inhibited by APAP^33^.

#### Oxidative stress response to acetaminophen cytotoxicity

**Figure 5:**
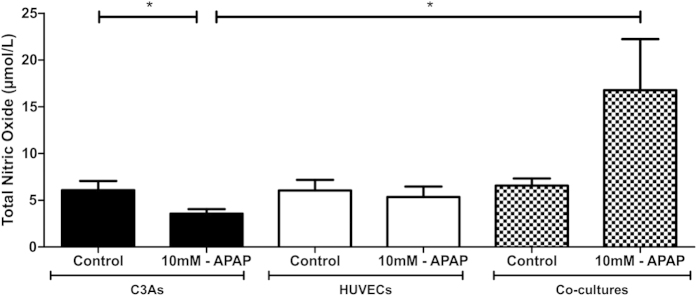
Oxidative stress in C3As and HUVECs mono- and co-cultures after 24 h of APAP treatment. Assessment of nitric oxide output in C3A (black bars) and HUVEC (white bars) mono-cultures or C3A/HUVEC co-cultures (patterned bars) following treatment with 0 mM (untreated control) or 10 mM APAP for 24 h. Data is expressed as the mean ± SEM (n = 3), *p < 0.05.

Total Nitric oxide (NO) output was also evaluated in C3As and HUVECs after APAP exposed at 24 h, as a potential indicator of reduced oxidative stress. NO concentration was significantly reduced in APAP-treated C3As in mono-cultures compared with untreated C3As. In contrast, compared with APAP-treated C3A mono-cultures, NO output in co-culture was significantly increased (p < 0.05; Fig. 5).

## Discussion

Organotypic *in vitro* human hepatic models for hepatotoxicity studies that more closely resemble *in vivo* hepatic functionality, could help facilitate improved strategies for early safety risk assessment during drug development. Herein, we demonstrate cell-type specific phenotypic stability and enhanced differentiated functionality in hepatocyte-like C3A cells, including CYP3A4 activity and albumin synthesis, when in co-culture with HUVECs (Figs 1 and 2). In agreement with *in vivo* studies^34^,^35^,^36^, acetaminophen toxicity was more profound in endothelial cells (Fig. 3D), compared with C3A mono-cultures (Fig. 3A–C); whilst in co-culture, particularly HUVECs appeared less phenotypically susceptible to the toxic effects of APAP (Fig. 3E). This resistance to APAP is also observed in a micropatterned PHH-fibroblast co-culture system at 30 mM APAP^6^,^7^. This simple and easy-to-implement human co-culture model may represent an alternative cell system to PHHs for initial hepatotoxicity screening or mechanistic studies of candidate compounds differentially targeting hepatocytes and endothelial cells.

Traditional strategies for assessing drug safety have low predictive capacity and may be overly precautionary^30^. Current *in vitro* toxicity assays based on hepatocyte monolayer cultures commonly give false-positives, with monocultures ‘over-sensitive’ to cytotoxic agents than in more ‘organotypic’ co-culture systems. As such, potentially safe and efficacious candidate compounds may be removed unnecessarily from drug development programmes.

Recent studies further highlight the need for improved organotypic liver culture models. Interspecies differences in APAP sensitivity are evident^37^. Whilst homotypic cultures of PHHs, hepatic cell lines (Huh 7; HepaRG) and iPSC-derived hepatocyte-like cells, show critical differences in mechanistic profiles in response to drugs (including APAP), involving widely-varying cellular ATP levels; and mode of cell death^38^. Given chemicals that have complex mechanisms of toxicity involving metabolic activation, such as APAP, co-culture systems may improve biological relevance by providing more *in vivo*-like complex adaptive responses to chemical exposure^39^.

The utility of the C3A:HUVEC co-culture as an organotypic liver culture model is discussed in the context of functionality and cytotoxic perturbations, increased resistance to APAP, endothelial cells (ECs) as early targets for hepatic toxicants; and the potential for early safety risk assessment and as an *in vitro* tool for aspects of mechanistic toxicology.

To assess the utility of co-cultures for metabolism studies, we demonstrated enhanced CYP3A4 activity and metabolic competency (albumin synthesis) compared with mono-cultures, and phenotypic stability (Figs 1 and 2). Overall, this demonstrated relevance of C3A:HUVEC co-cultures for evaluating drug metabolism and toxicity^6^,^7^,^40^; and compared well with current *in vitro* organotypic liver culture systems (SI Table S1).

To identify a potential risk, many pharmaceutical companies incorporate some combination of cytotoxicity parameters as in the present study, including: oxidative stress, ATP depletion, altered redox homeostasis, and impaired respiration^41^.

When C3As were co-cultured with HUVECs and exposed to APAP doses of 5 mM or 10 mM, cells exhibited significantly higher resistance to APAP toxicity, maintaining intracellular ATP content, with reduced oxidative stress and mitochondrial toxicity, compared with mono-cultures (Fig. 3). This implies that interactions between C3As and HUVECs may be the result of different mechanisms of drug toxicity in co-culture *vs* mono-cultures. ATP is typically reduced following APAP challenge [≥10 mM] in mono-cultures^38^, although cells can increase ATP content preceding apoptosis^42^.

Recent modeling strategies of human hepatotoxicity, including sophisticated micropatterning techniques with PHHs-fibroblasts, hepatocyte-EC cell sheet engineering and iPSC-derived hepatocytes with endothelial cells have demonstrated enhanced hepatic activity^2^,^15^,^16^,^24^,^25^,^26^,^43^; and responsiveness to drugs, similar to our system (see also SI Table S1). Co-culture models permit complex heterotypic cell-cell/cell-matrix interactions, which allow bidirectional communication - essential to provide a closer representation of functional homeostasis of the liver and *in vivo* drug metabolism^15^,^16^,^17^.

The C3A:HUVEC co-culture model may also provide insight for the investigation of mechanistic aspects of drug toxicity. We used APAP as a model hepatotoxin, which follows the 3-step model of DILI (Willett *et al.* ref. 27). The first step, as an example, might include direct cell stress (cytotoxicity) or direct mitochondrial inhibition (Fig. 3B); the second step may involve mitochondrial dysfunction, such as by disruption of the permeability transition pore (PTP), which determines the extent of ATP depletion (Fig. 3A,B); the third step, essentially determines the type of cell death: necrosis (greater depletion of ATP) or apoptosis (a lesser depletion of ATP; caspase activation by cytochrome c requires ATP) [Kaplowitz – Ed. 2013: ISBN: 978-0-12-387817-5]. In mono-cultures, the reduction in ATP with commensurate changes in membrane integrity (cytotoxicity) (Fig. 3B), indicates that APAP is a mitochondrial toxin at an intermediate threshold dose of 10 mM APAP; with apparent (significant) primary necrosis also taking place at 5 mM APAP. In co-cultures, the trend was greater resistance to mitochondrial toxicity and increased sensitivity to necrosis at all doses tested, compared with C3A and HUVEC mono-culture controls. Given this assay designed to predict potential mitochondrial dysfunction as a result of xenobiotic exposure, initial cytotoxicity screening utilizing organotypic co-cultures, may inform subsequent drug screening strategies for complex adverse outcomes, and provide valuable information on whether to perform more stringent assays of mitochondrial dysfunction/mode of cell death, such as: Cytochrome c release (apoptosis marker), reactive oxygen species (ROS) production, mitochondrial protein-APAP adduct formation; or the M65 necrosis marker. Indeed, NAPQI-independent targeting of mitochondrial complex III might be responsible for acetaminophen toxicity in extra-hepatic tissues^27^. Thus use of surrogate extra-hepatic endothelial tissue surrogates may help reveal alternative (eg Stage-II) mechanisms involved in APAP toxicity.

### Lactate as an oxidative stress biomarker

The observed resistance to toxicity in co-cultures is further supported by the finding of lower lactate levels in co-culture supernatants, compared with HUVEC mono-cultures (Fig. 4), indicative of reduced oxidative stress^32^. *In vivo,* levels of lactate rise due to endothelial damage, which results in the accumulation of erythrocytes within the space of Disse; again suggesting the endothelium as an early target in APAP overdose^28^,^35^,^44^,^45^,^46^. The significantly higher lactate levels seen in HUVEC monolayers suggest endothelial cells as the primary source of lactate released under APAP toxicity in this co-culture model system. Further studies are needed to elucidate the role of the heterotypic cell interactions in reducing cytotoxicity and involvement with known signaling pathways involved in oxidative stress.

### Cellular Redox Changes

We observed a switch favoring aerobic metabolism (P/L ratio > 1) in the presence of excess NAC following exposure to APAP in both C3As and HUVECs monolayers (Fig. 4). Given ‘normal’ C3A metabolism is anaerobic (Patent No. WO 1991018087 A1; 1991), excess NAC may enhance the pool of cysteine which is then degraded and ends up as an energy substrate in the Krebs cycle to support oxidative phosphorylation, resulting in enhanced mitochondrial energy (ATP) as well as pyruvate production. However, although the P/L ratio in co-culture remained <1 (anaerobic), at the same time as they presented high ATP content and resistance to APAP toxicity, this is perhaps not surprising given NAC has little physiological effect under normal ‘organotypic’ conditions^47^. Thus the combination of elevated ATP and pyruvate levels in our co-culture model may effectively provide a hepato-protective environment through enhanced GSH activity against APAP toxicity; GSH acting as a major scavenger of ROS and peroxynitrite^48^,^49^. We show that NAC appears to significantly enhance pyruvate in co-culture (Fig. 4). Interestingly, pyruvate has antioxidant and free radical scavenger properties^50^, whilst addition of energy substrates, such as pyruvate to rat hepatocyte cultures, has been reported to maintain hepatic-specific albumin production and both bile canaliculi and the cytosolic phase II biotransformation enzyme glutathione-S-transferase^51^. Taken together, these observations could explain some of the apparent cytoprotective effects of NAC through enhancement of ATP and antioxidant potential.

### Endothelial cellular response to APAP

Metabolically competent HUVECs constitutively produce CYP2E1, at least at the transcriptional level. Therefore, the observed effective ‘resistance’ to APAP toxicity in co-culture could also in part be due to an enhanced capacity to process APAP activation/metabolism, including both C3A and endothelial cellular response to injury. APAP toxicity is greater for HUVECs than C3As in mono-culture, In fact this greater toxicity of APAP against HUVECs is consistent with emerging evidence that both HUVECs and LSECs may possess a significant ability to metabolize APAP and that LSECs are an early target for hepatic toxicants, prior to any evidence of parenchymal cell injury^34^,^35^,^36^. CYP450 enzymes in vascular endothelial cells may play important roles in determining metabolic fates of circulating pro-toxicants^52^. Further work using our co-culture model could involve determining basal CYP450 protein expression levels and in response to hepatotoxins.

### Nitric Oxide

APAP toxicity in C3A:HUVEC co-cultures (Fig. 5), was associated with increased levels of total NO which can also increase intracellular GSH^53^, inferring protection from oxidative stress^54^. This may have important implications for identifying redox-sensitive cell signaling pathways that can be activated by NO. NO can be produced in both endothelial cells and hepatocytes and has been reported to have both cytoprotective effects and deleterious effects (increased ROS production; fat accumulation in hepatocytes), depending on the stressor and culture system^48^. That NO functions as a purely cytoprotective effector molecule has been proposed, as it has a significant barrier to chemical reduction, so preventing endogenous formation of the potentially toxic reduced species (HNO/NO–)^55^. The observed complex effects may also be in part due to the fact that the reactivity of NO *per se* may be overestimated *in vitro*, because no drain is provided to remove NO. During APAP hepatotoxicity, peroxynitrite (superoxide + NO = peroxynitrite (ONOO−), a highly reactive, potent oxidant and nitrating species is generated in hepatocytes and sinusoidal endothelial cells, and can cause mitochondrial damage and oxidative cellular stress^54^. However, increased NO levels have been observed in AML-12 hepatocytes exposed to a NO donor with concomitant activation of antioxidant transcription factors^56^. Given increased NO levels were seen in APAP-resistant co-cultures (Fig. 5), we may also speculate that NO could induce an elevation of reduced glutathione (GSH)^54^. Moreover, NO production was dramatically increased in APAP-toxic mice^57^; whereas inhibition of cytokine-mediated NO production potentiates APAP hepatotoxicity by modulation of hepatocyte glutathione stores - suggesting our organotypic model may mimic certain *in vivo* parameters of toxicity^58^.

It is therefore feasible that co-culture facilitates cross-talk between C3A-HUVECs, which enhances NO production, which in our system confers an hepatoprotective environment in response to APAP. We have also found that VEGFR-2 expression was significantly greater in APAP-treated co-cultures compared with untreated controls (data not shown). Indeed, VEGF levels increased 30× fold in an APAP-treated mouse model, conferring hepatotoprotective effects, with concomitant increase in VEGFR-2 expression^57^.

Recent studies have shown that HepG2 mono-cultures treated with 20 mM APAP showed no evidence of oxidative stress, mitochondrial dysfunction, or cell injury; suggesting very low levels of drug-metabolizing enzymes, radically reducing APAP activation thus preventing any toxicity in HepG2 cells^48^,^59^; which are commonly used for studies of hepatotoxicity mechanisms and screening of potential DILI compounds^38^. In contrast, homotypic C3A cell cultures were dose-responsive to APAP (Fig. 3A). Despite significantly enhanced differentiated function (CYP3A4 activity/albumin production), – a feature of more differentiated/stabilized cultures) in co-culture, resistance to APAP toxicity remains. This adaptive response may involve bidirectional communication between C3As and ECs, which may alter classic APAP toxicity pathways. Given the importance of cell-cell/cell-matrix interactions in maintaining differentiated phenotype through reciprocal signalling^26^, further mechanistic studies could confirm whether cell adhesion molecules, such as integrins, may mediate the physical heterotypic interactions between hepatocytes and ECs^24^,^26^.

## Conclusions

We have developed an organotypic human hepatic *in vitro* model as a pre-clinical hepatotoxicity tool, which could better inform and be complementary to animal testing, for early safety risk assessment. Co-culture maintained hepatic- and endothelial-specific phenotype with enhanced hepatic functional activity. We have demonstrated that C3A cells (mono-cultures) do show an enhanced degree of APAP toxicity albeit less than HUVECS, while co-culture with HUVECs shows enhanced resistance despite enhanced CYP3A4 activity and albumin production - features of a more differentiated phenotype. Resistance to APAP toxicity in co-culture may also be a result of enhanced production of cellular ATP, NO and GSH; as compared with mono-cultures of C3As or ECs. Profound cytotoxic effects of APAP on ECs, which concur with *in vivo* findings, warrant further investigation. The complex interplay observed in heterotypic co-culture may better reflect the situation found *in vivo* by providing a more physiologically-relevant *in vitro* tool, and as a paradigm for insight into mechanisms of drug metabolism and toxicity.

## Additional Information

**How to cite this article**: Nelson, L. J. *et al.* Acetaminophen cytotoxicity is ameliorated in a human liver organotypic co-culture model. *Sci. Rep.* **5**, 17455; doi: 10.1038/srep17455 (2015).

## Supplementary Material

Supplementary Information
