# Supplementary material for: Acetaminophen cytotoxicity is ameliorated in a human liver organotypic co-culture model
Source: Sci Rep. 2015 Dec 3;5:17455. doi: 10.1038/srep17455 (PMC4668374; doi:10.1038/srep17455)
Supplement: Supplementary Information [file srep17455-s1.pdf]

Supplementary Information

Acetaminophen cytotoxicity is ameliorated in a human liver organotypic co-culture model

*†Leonard J Nelson[1] †Maria Navarro[1], Philipp Treskes[1], Kay Samuel[2], Olga Tura-Ceide[3], Steven D Morley[1], Peter C Hayes[1] and John N Plevris[1]

Supplementary Information Table S1 Hepatic-specific function in different in vitro culture systems compared with primary human hepatocytes

**Culture system**<br>**(2D format unless stated)**	**Albumin**<br>**μg/hr/million cells**<br>**(unless stated)**	**CYP450s: CYP3A4 unless stated (Activity/ Inducibility/ Functionality)**	**Response to Acetaminophen (APAP) Toxicity**	**References / Comment**
Primary human Hepatocytes (PHHs) (mono-culture)	0.05-0.8 (subject to donor variability)	High	Yes (sensitive)	(Chesne 1993, Lübberstedt, Müller-Vieira et al. 2011)
PHHs (mono-culture)<br>Micropatterned cocultures (PHHs:mouse fibroblasts)	0.01 (d3)<br>0.5 (d1)<br>1.0 (d3)	High	Yes (sensitive)	(Khetani and Bhatia 2008)
PHHs (mono-culture)<br>PHHs:HUVECs co-culture	0.001 (d3)<br>0.003 (d3)	CYP3A4 (diazepam metabolites)	ND	(Salerno, Campana et al. 2011)
PHH-MPCCs (PHH-based micro-patterned co-cultures containing mouse 3T3-J2 fibroblasts)	6-7 (> in vivo rates) (subject to donor variability)	High Transcriptome comparable with fresh PHHs; & PHH-MPCCs	Yes (sensitive to multiple dosing)	(Berger, Ware et al. 2015)
C3A monoculture	*1.0	ND	ND	(Filippi, Keatch et al. 2004)
**C3A monoculture**<br>**Co-culture (C3A:HUVECs)**	**0.83**<br>**1.42**	**Low drug metabolizing enzymes (DMEs) (2.5x fold increase in co-culture)**	**Yes (low in C3A mono-culture); High sensitivity in HUVEC mono-cultures**	**Present study (see Fig. 2)**
HepG2 mono (static)<br>Co-culture (static) (HepG2:HUVECs)<br>HepG2 mono (bioreactor)<br>Co-culture (bioreactor)	0.01<br>0.03<br><br>0.015<br>0.05	Very low (DMEs) in mono	No (insensitive)	(Guzzardi, Vozzi et al. 2009)

Rat hepatocytes monoculture<br>Hepatocytes/LSEC co-culture<br>hepatocytes/LSEC/fibroblast tri-culture	3.0 μg/day<br>1.0 μg/day<br>11.0 μg/day	ND	ND	(March, Hui et al. 2009)
Rat hepatocytes monoculture<br><br>Co-culture<br>Rat hepatocytes:bovine carotid artery (ECs)	0.5 (d1);<br>0.2 (d7)<br>0.6 (d1)<br>1.0 (d7)	ND	ND	(Kim, Ohashi et al. 2012)
HepaRG co-culture 2D (3D)<br>(Hepatocytes:cholangiocytes)<br>2D*	0.21 (0.49)<br><br>*0.15	High (120% of PHH value)	Yes (Sensitive)	(Lübberstedt, Müller-Vieira et al. 2011, Leite, Wilk-Zasadna et al. 2012)
In vivo values:<br>(human liver)<br>(rat liver)	 2.0<br>2.4	 184*<br>292*<br>[Total P450 content<br>(pmol/ mg TP)	Yes (sensitive)	(Gómez-Lechón, López et al. 1990, Lazar, Mann et al. 1995, Donato, Castell et al. 1999, Iwata, Sajiki et al. 1999)
HUVECs	ND	CYP2E1 transcripts' constitutive expression	ND	(Farin 1994)

Table S1 Hepatic-specific function/ expression in different in vitro culture systems compared with primary human hepatocytes, in vivo (liver) data from different species and HUVECs. Several factors including culture protocols (eg cell type/ species used) hepatic functions, culture conditions and duration vary greatly between research groups makes direct comparisons difficult. For clarity, mean values are shown. In vivo values are shown for human and rat livers. Abbreviations: ND, not determined in referenced study. ND, Not determined or not measured.

References

Berger, D. R., B. R. Ware, M. D. Davidson, S. R. Allsup and S. R. Khetani (2015). "Enhancing the functional maturity of induced pluripotent stem cell-derived human hepatocytes by controlled presentation of cell-cell interactions in vitro." Hepatology **61**(4): 1370-1381.

Chesne, C. (1993). "Viability and function in primary culture of adult hepatocytes from various animal species and human beings after cryopreservation*1." Hepatology **18**(2): 406-414.

Donato, M. T., J. V. Castell and M. J. Gómez-Lechón (1999). "Characterization of drug metabolizing activities in pig hepatocytes for use in bioartificial liver devices:

comparison with other hepatic cellular models." Journal of Hepatology **31**(3): 542-549.
Farin, F. (1994). "Expression of Cytochrome P450s and Microsomal Epoxide Hydrolase in Primary Cultures of Human Umbilical Vein Endothelial Cells." Toxicology and Applied Pharmacology **124**(1): 1-9.
Filippi, C., S. A. Keatch, D. Rangar, L. J. Nelson, P. C. Hayes and J. N. Plevris (2004). "Improvement of C3A cell metabolism for usage in bioartificial liver support systems." J Hepatol **41**(4): 599-605.
Gómez-Lechón, M. J., P. López, T. Donato, A. Montoya, A. Larrauri, P. Giménez, R. Trullenque, R. Fabra and J. V. Castell (1990). "Culture of human hepatocytes from small surgical liver biopsies. Biochemical characterization and comparison with in vivo." In Vitro Cellular & Developmental Biology **26**(1): 67-74.
Guzzardi, M. A., F. Vozzi and A. D. Ahluwalia (2009). "Study of the Crosstalk Between Hepatocytes and Endothelial Cells Using a Novel Multicompartmental Bioreactor: A Comparison Between Connected Cultures and Cocultures." Tissue Engineering Part A **15**(11): 3635-3644.
Iwata, H., T. Sajiki, H. Maeda, Y. G. Park, B. Zhu, S. Saioh, T. Uesugi, I. Ikai, Y. Yamaora and Y. Ikada (1999). "In Vitro Evaluation of Metabolic Functions of a Bioartificial Liver." ASAIO Journal **45**(4): 299-306.
Khetani, S. R. and S. N. Bhatia (2008). "Microscale culture of human liver cells for drug development." Nat Biotechnol **26**(1): 120-126.
Kim, K., K. Ohashi, R. Utoh, K. Kano and T. Okano (2012). "Preserved liver-specific functions of hepatocytes in 3D co-culture with endothelial cell sheets." Biomaterials **33**(5): 1406-1413.
Lazar, A., H. J. Mann, R. P. Remmel, R. A. Shatford, F. B. Cerra and W.-S. Hu (1995). "Extended liver-specific functions of porcine hepatocyte spheroids entrapped in collagen gel." In Vitro Cellular & Developmental Biology - Animal **31**(5): 340-346.
Leite, S. B., I. Wilk-Zasadna, J. M. Zaldivar, E. Airola, M. A. Reis-Fernandes, M. Mennecozzi, C. Guguen-Guillouzo, C. Chesne, C. Guillou, P. M. Alves and S. Coecke (2012). "Three-Dimensional HepaRG Model As An Attractive Tool for Toxicity Testing." Toxicological Sciences **130**(1): 106-116.
Lübberstedt, M., U. Müller-Vieira, M. Mayer, K. M. Biemel, F. Knöspel, D. Knobeloch, A. K. Nüssler, J. C. Gerlach and K. Zeilinger (2011). "HepaRG human hepatic cell line utility as a surrogate for primary human hepatocytes in drug metabolism assessment in vitro." Journal of Pharmacological and Toxicological Methods **63**(1): 59-68.
March, S., E. E. Hui, G. H. Underhill, S. Khetani and S. N. Bhatia (2009). "Microenvironmental regulation of the sinusoidal endothelial cell phenotype in vitro." Hepatology **50**(3): 920-928.
Salerno, S., C. Campana, S. Morelli, E. Drioli and L. De Bartolo (2011). "Human hepatocytes and endothelial cells in organotypic membrane systems." Biomaterials **32**(34): 8848-8859.
